# Surgical approaches to canine appendicular osteosarcoma part 1- anatomic landmarks and amputation techniques

**DOI:** 10.3389/fvets.2025.1655764

**Published:** 2025-11-18

**Authors:** Ryshely Sonaly De Moura Borges, Paloma Helena Sanches da Silva, Pedro Antônio Bronhara Pimentel, Renato Dornas de Oliveira Pereira, Angel Almendros, Antonio Giuliano, Rodrigo dos Santos Horta, Paulo Vinícius Tertuliano Marinho

**Affiliations:** 1 Department of Veterinary Medicine and Surgery, Veterinary School, Universidade Federal de Minas Gerais, Belo Horizonte, Brazil; 2 Department of Veterinary Clinical Sciences, Jockey Club College of Veterinary Medicine, City University of Hong Kong, Kowloon, Hong Kong SAR, China; 3 Harvest Veterinary Oncology Center, Kowloon, Hong Kong SAR, China; 4 Department of Veterinary Medicine, Instituto Federal Sul de Minas Gerais, Muzambinho, Brazil

**Keywords:** appendicular skeleton, bone tumor, oncological surgery, oncology, surgical anatomy

## Abstract

Osteosarcoma (OSA) is the most common primary bone neoplasm affecting dogs and the appendicular bones are frequently affected, accounting for up to 80% of reported cases. After tumor diagnosis and staging, surgery followed by adjuvant chemotherapy is the standard treatment of care. The purpose of this narrative literature review is to describe the anatomical landmarks and amputation techniques performed in the treatment of canine appendicular OSA, also using cadaveric models to demonstrate it. Surgical treatment options may include amputation of the affected limb, considered the standard of care. For thoracic limbs anterior quarter amputation, amputation with shoulder disarticulation, and midhumeral amputation. For pelvic limbs, amputation with hemipelvectomy, amputation with hip disarticulation, and midfemoral amputation. Anatomical knowledge is fundamental for performing a meticulous and correct technique, which allows a lower risk of recurrence and intra-operative and post-surgical complications.

## 1 Introduction

The most prevalent bone neoplasm in dogs is osteosarcoma (OSA). The incidence of OSA is higher in large to giant dog breeds, which often complicates clinical and therapeutic management, although it can also affect medium and small breed dogs and cats (1, 63). Given the highly aggressive nature of this neoplasia, especially in the canine species, in which the prognosis can range from guarded to poor, immediate surgical intervention is recommended to achieve local control (2).

For appendicular OSA, due to its locally aggressive behavior, the surgical procedure prioritizes local control through wide or radical excisions (3–6). Limb amputation remains the most frequently performed radical technique. Radical excision involves complete removal of the anatomical compartment or structure of origin, including techniques such as amputation (7).

While effective for local disease control, it may directly impact the patient's quality of life, particularly in cases with concurrent articular/neuromusculoskeletal comorbidities or obesity (2, 8). Therefore, the patient's overall condition, tumor aggressiveness, and lesion location should guide the surgical plan, as these factors play a crucial role in determining the prognosis (3, 8).

To perform limb amputation techniques in the treatment of OSA, knowledge of the topographic anatomy of the bones, joints, ligaments, tendons, muscles, blood vessels, and nerves of the limb are essentials to avoid unnecessary dissection and transection. Therefore, this article aims to describe the radiosurgical approaches for the treatment of appendicular OSA in dogs, based on current best practices and evidence, with particular emphasis on relevant anatomical considerations. For this purpose, canine cadavers were used.

## 2 Literature review

### 2.1 Canine osteosarcoma

OSA is a primary malignant bone neoplasm that affects both dogs and other vertebrate species, including humans. This cancer is characterized by the proliferation of osteoblastic mesenchymal cells with abundant osteoid matrix production. It is up to 50 times more frequent in dogs, although its true incidence remains poorly characterized (9). Anatomically, OSA in dogs primarily affects the skeletal system, including both the axial and appendicular skeleton (9, 10), with the appendicular skeleton accounting for 70–80% of cases (11). The thoracic limbs are more frequently affected than the pelvic limbs, potentially related to their greater weight-bearing role and subsequent bone stress (12). OSA may also arise in extraskeletal sites, such as the spleen and mammary glands, though such cases are rare (9, 13). In long bones, the metaphyseal region is the predominant site for tumor development, particularly the proximal humerus, distal femur, and proximal or distal tibia (1, 10, 12, 63).

The epidemiology of appendicular OSA exhibits distinct species-specific patterns between humans and canines. In humans, OSA is a rare malignancy with a peak incidence during adolescence (14). Conversely, in dogs, OSA demonstrates a higher prevalence among elderly populations and represents one of the most frequently diagnosed tumors in large- to giant-breed dogs (1). It is most commonly diagnosed in large to giant dog breeds, including Saint Bernard, Great Dane, Irish Setter, Irish Wolfhound, Doberman Pinscher, Rottweiler, German Shepherd, and Golden Retriever (1, 63). Notably, both species exhibit a bimodal age distribution, with disease onset observed in both juvenile and geriatric individuals (1, 14). In dogs, the disease exhibits a bimodal age distribution, with incidence peaks in middle-aged to elderly dogs (7–9 years) and young dogs (<3 years) (9).

The primary tumor causes severe bone destruction, periosteal reaction and adjacent soft tissue invasion and swelling, leading to pain, lameness, and potential pathological fractures (1, 63). Its biological behavior is highly aggressive, with metastatic potential exceeding 90% in dogs (2, 9). Diagnosis is based on clinical history, lameness, localized limb swelling, radiographic evidence of bone lysis or destruction, and cytological or histopathological evaluation. Definitive confirmation requires histopathological analysis (1). Staging involves imaging to assess pulmonary metastasis. Thoracic radiography (sensitivity: 71–95%) or computed tomography (CT) may be used, with CT being more sensitive, particularly in large to giant breeds (2, 15).

The management of appendicular OSA has seen limited progress over recent decades, with therapeutic strategies remaining largely unchanged. Current standard-of-care approaches include surgical resection or radiotherapy when available and adjuvant chemotherapy such as cisplatin or carboplatin (2, 7, 16, 17). Given the high rate of distant metastasis, systemic chemotherapy remains essential to improve overall survival and should be initiated as early as possible after surgery. The timing of chemotherapy has been identified as a prognostic factor, as dogs receiving adjuvant treatment within 5 days after amputation achieved a median survival time of 375 days compared with 202 days in those that started later (*p* = 0.005) (18).

Surgery is the first-line treatment to remove the primary tumor and provide analgesic relief. Approaches include radical techniques (e.g., limb amputation) and limb-sparing procedures such as scapulectomy (11, 16), partial ulnectomy, bone segment excision followed by reconstruction using cortical allografts, metallic endoprostheses (8, 61), endo-exoprostheses (19), pasteurized tumor autografts (20), ulnar rollover transposition (4), limb shortening (62), or bone transport distraction.

Emerging approaches leveraging immunotherapy and targeted therapies represent a promising frontier in OSA research (21). A deeper understanding of the tumor microenvironment and host immune response may enable the development of treatments that inhibit immune checkpoints and proteins critical to tumor progression and metastasis (22). Novel immunotherapies, including immune checkpoint inhibitors, dendritic cells vaccines, chimeric antigen receptor T cells, and specific antibodies are currently undergoing preclinical evaluation *in vitro* and in early-phase clinical trials (21, 22). Despite promising research, canine oncology lags significantly in the development of commercially available therapies that could improve OSA clinical outcomes in dogs, with minimal advancements over the past decades (23).

### 2.2 Key anatomical landmarks of the thoracic and pelvic limbs in dogs

According to the Nomina Anatomica Veterinaria (60, 64), the canine thoracic limb, a component of the appendicular skeleton, comprises four segments: the scapular girdle (*cingulum membri thoracici*, formed by the scapula and clavicle, the latter being vestigial or absent in most dogs), the brachium (*skeleton brachii*, arm, represented by the humerus), the antebrachium (*skeleton antebrachia*, forearm, consisting of the radius and ulna), and the *manus* (*skeleton manus*, paw, including the carpus, metacarpus, digits with phalanges, and associated sesamoid bones). Beyond osseous structures, intrinsic musculature—originating and inserting within the limb itself—plays a critical role in facilitating articular movements.

In the lateral scapular and shoulder region, the *deltoideus* muscle (comprising spinal and acromial portions) lies superficially, overlaying the deeper *supraspinatus* and *infraspinatus* muscles, which run parallel to the scapular spine. The medial scapular and shoulder region is characterized by the *teres major, subscapularis*, and *coracobrachialis* muscles. Caudal to the humerus, the *triceps brachii* muscle (with long, lateral, medial, and accessory heads), *anconeus* muscle, and *tensor fasciae antebrachii* muscle are situated, while cranial to the humerus, the *biceps brachii* and *brachialis* muscles are prominent.

The craniolateral *antebrachium* includes the *extensor carpi radialis*, common digital extensor, lateral digital extensor, lateral ulnar, supinator, and *abductor pollicis longus* muscles (24). Conversely, the caudomedial *antebrachium* features the *flexor carpi radialis*, superficial digital flexor, deep digital flexor, *flexor carpi ulnaris, pronator teres*, and quadrate pronator muscles (66). These muscles collectively enable precise limb mobility, joint stabilization, and force transmission during locomotion.

The nerves that innervate the thoracic limb originate from the brachial plexus, which courses caudoventrally between the *subscapularis, supraspinatus*, and *serratus ventralis* muscles. Adjacent to the brachial plexus on the medial aspect of the limb lies the axillary artery, which branches into the subscapular artery (along the caudal scapular border) and the circumflex scapular artery (near the shoulder joint). The axillary artery continues as the brachial artery, running along the medial brachium and maintaining proximity to the musculocutaneous, radial, median, and ulnar nerves. Distally, the brachial artery transitions into the median artery within the antebrachium. The median artery gives rise to the radial artery, which follows the caudomedial limb margin, and the ulnar artery, which courses laterally and distally alongside the ulnar nerve. These arteries further branch into dorsal and palmar carpal arteries (66).

The pelvic limb of dogs is composed of four segments: the pelvic girdle is formed by the coxal bone (ilium, pubis, and ischium), including the acetabulum (hip joint), sacroiliac joint, and pelvic symphysis (pubic and ischial symphyses); the thigh is represented by the femur and patella; the leg comprises the tibia and fibula; and the foot includes the tarsus, metatarsus, and digits (phalanges and associated sesamoid bones).

The intrinsic musculature of the pelvic limb, essential for articular motion, is organized regionally. The lateral hip muscles include the *tensor fasciae latae, gluteus medius*, and *gluteus profundus* (25, 26). The caudal hip is composed of the internal obturator, external obturator, *gemelli*, and *quadratus femoris* muscles. In the cranial thigh, the iliacus and quadriceps femoris (*rectus femoris, vastus medialis, vastus lateralis*, and *vastus intermedius*) play key roles. The caudal thigh comprises the *biceps femoris, semitendinosus*, and *semimembranosus* muscles, while the medial thigh includes the *sartorius, gracilis, pectineus*, and abductor muscles (25, 26). The leg musculature is divided into cranial and caudal groups: the cranial muscles consist of the cranial tibial, long fibular, short fibular, long digital extensor, and lateral digital extensor, whereas the caudal muscles include the *gastrocnemius, popliteus*, superficial digital flexor, and deep digital flexor (25, 27).

The extrinsic musculature of the pelvic limb consists of the superficial gluteal, *piriformis*, and psoas major muscles, which are responsible for movement and stabilization by connecting the limb to the trunk (66). On the medial aspect of the pelvic limbs, there is a small anatomically significant region known as the femoral triangle, named for its geometric shape. This structure lies beneath the deep medial femoral fascia and is bordered cranially by the caudal edge of the sartorius muscle and caudally by the cranial border of the *pectineus* muscle. Within this key anatomical landmark are the femoral artery, the primary arterial supply to the pelvic limb, which is positioned cranially to its corresponding vein, and the saphenous nerve, a branch of the femoral nerve that may be located cranial or lateral to the femoral artery (25). Proximally, the iliopsoas muscle is found, while the vastus medialis muscle lies in a deeper position.

The nerves that innervate the pelvic limb originate from the lumbosacral plexus. Among them, the sciatic nerve runs caudally to the greater trochanter of the femur, along the lateral aspect of the thigh, and distally branches into the tibial and common fibular nerves (25, 66).

### 2.3 Anatomy and surgical excision of peripherical lymph nodes of interest–brief description

Veterinary oncological surgery has been gaining increasing popularity, and lymphadenectomy in dogs and cats, previously rarely performed, has been reviewed in recent years due to its diagnostic and prognostic relevance in many neoplasms. However, despite being highly metastatic, OSA rarely spreads to the lymph node. In a study of appendicular OSA, lymph node metastasis was found in only 10/228 (4.4%) cases (28).

Although lymphadenectomy in dogs has gained prominence in the literature, there are still few surgical descriptions for peripheral lymph node excision, which likely results in increased operative time and increased tissue trauma due to difficulty identifying the structures of interest, and, consequently, increased overall risk associated with the procedure (29). Recently, some technical descriptions have been published detailing surgical approaches to optimize lymph node harvesting, particularly from the axillary and superficial inguinal lymph centers (30–33).

Worden et al. (29) compared lymphadenectomies of the afore mentioned lymph nodes using the technique based on superficial anatomical landmarks with the standard surgical approach and the methylene blue dye-guided approach, performed by novice surgeons on canine cadavers. The authors concluded that landmark-guided lymphadenectomy may be useful in reducing surgical time, tissue injury, and difficulty. The landmark-guided technique reduced the time to identify axillary lymph nodes, alleviated the subjective difficulty of superficial inguinal lymph node excision, and decreased tissue trauma during axillary and superficial inguinal lymphadenectomies (29).

With the exception of inguinal lymphadenectomy, routine limb amputation inherently includes popliteal lymphadenectomy (in cases of pelvic limb amputation via coxofemoral disarticulation or hemipelvectomy) and superficial axillary/cervical lymphadenectomy (in forequarter amputation including scapulectomy). Nevertheless, histological evaluation of the lymph nodes is important, as it helds prognostic information for canine OSA; therefore, the pathology request form should emphasize the inclusion and identification of the lymph node (28).

Lymph nodes draining the thoracic limbs include the principal axillary lymph node and the superficial cervical lymph nodes. The principal axillary lymph node (*Lnn. axillares proprii*) is located medially to the shoulder joint at the level of the first and second ribs, adjacent to the thoracodorsal artery and vein, axillary artery and vein and the brachial plexus, embedded in adipose tissue, in a location that is difficult to access due to the robust musculature of the thoracic limb, beneath the aponeurosis between the *latissimus dorsi* and deep pectoral muscles (31). The rate of presence of the accessory axillary lymph node varies in dogs, but is frequently low: 13.8% (present in 4 of 29 dogs) (34), 4.3% (4/90 dogs) (35), with a slightly higher incidence of 25% being reported (36). When present, the accessory axillary lymph node is attached to the medial and deep part of the latissimus dorsi muscle at the level of the third or fourth intercostal space (31). Those lymph nodes drains the muscles and tendons of the limb, excluding the shoulder, elbow, carpal, and phalangeal joints (37).

For axillary lymphadenectomy, the patient can be positioned in lateral recumbency, with the thoracic limb on the side to be accessed abducted and extended cranially to expose the axillary region at the level of the first intercostal space. The region between the dorsal border of the superficial pectoralis muscle and the ventral border of the latissimus dorsi muscle can be identified as a palpable depression in the caudal axillary region and close to the ipsilateral lateral chest wall. After a 3-cm skin incision, starting approximately 6 cm caudal to the caudal border of the triceps brachii, in the direction of the division of the first identified muscles, the cutaneous muscle of the trunk is divulsed, and the intersection between the latissimus dorsi and superficial pectoralis muscles is separated with blunt dissection. By separating the fascia that connects them, the thoracodorsal nerve is visualized, and immediately dorsal to it is the main axillary lymph node, surrounded by adipose tissue and adjacent to the axillary artery and vein (31). The surrounding adipose tissue is dissected to visualize the main axillary lymph node, followed by hemostasis of the afferent and efferent vessels with absorbable monofilament suture (size 4-0). The latissimus dorsi muscle can be elevated and reflected through the incision to expose its deepest portion; when present, the accessory axillary lymph node is attached to this more ventral part, within the musculature itself or surrounded by fat, but caudal to the main lymph node and can also be excised. The surgical site is closed with myorrhaphy of the latissimus dorsi muscle, deep pectoralis and latissimus dorsi, followed by subcutaneous tissue suture and then dermorrhaphy (30–32).

As an alternative to the open technique, considering reduced incision size and dissection, in addition to the absence of the need for aggressive muscle retraction, Kuvaldina et al. (31) developed and described a minimally invasive endoscopic technique for excisional biopsy of axillary lymph nodes in dogs. The authors conclude that the endoscopic technique is feasible and can be used in patients in whom an excisional biopsy of the axillary lymph node is necessary.

The superficial cervical lymph nodes (*Lnn. cervicales superficiales*) lie deep to the *brachiocephalicus* muscle, cranial to the *supraspinatus* muscle and superficial cervical artery, along the prescapular branch. These nodes drain the antebrachium, carpus, metacarpus, and digits (37). In superficial cervical lymphadenectomy, the patient is positioned in lateral recumbency, with the neck extended and the forelimbs directed caudally. A skin incision is made 3.0 cm cranial to the scapulohumeral joint, at the level of the acromion. After divulsion of the subcutaneous tissue, the omotransversus muscle is identified, and medial to it, the lymph node can be exposed for removal. The procedure is completed with synthesis of the subcutaneous tissue and skin (38, 39).

The lymph nodes responsible for draining the pelvic limbs include the sacral, popliteal, occasionally the medial femoral, and the superficial inguinal lymph nodes. The sacral lymph nodes are located within the pelvic cavity and drain the muscles of the ischiatic region of the pelvis. The superficial popliteal lymph node represents the only lymph node in the popliteal lymphatic center, is situated on the caudal surface of the *gastrocnemius* muscle, specifically within the popliteal fossa between the *biceps femoris* and *semitendinosus* muscles, with the lateral saphenous vein adjacent (40). It drains the lateral regions of the knee joint, tibia, tarsus, metatarsus, and phalanges. When present, the medial femoral lymph node is found on the medial aspect of the thigh, adjacent to the caudal border of the femoral vessels, and may contribute to the drainage of the medial regions also drained by the popliteal lymph node (37).

For popliteal lymphadenectomy, the patient may be in the lateral recumbency position for a skin incision in the caudal region of the knee joint (38, 39). Immediately after opening the skin plane and divulsing the subcutaneous tissue, the adipose tissue containing the lymph node is visualized and then dissected for excision (38, 39). The technique ends with the synthesis of the accessed tissue planes.

Dogs have up to four superficial inguinal lymph nodes, which form part of the inguinofemoral lymph center. These lymph nodes are located caudal to the inguinal mammary glands, approximately 1.0 cm from the linea alba, between the dorsolateral penile edge and the level of the bulbus glandis in males (32, 40). They are responsible for draining the caudal portion of the pelvis, the lateral and medial aspects of the thigh, as well as the craniomedial regions of the knee joint, tibia, tarsus, metatarsus, and digits (37).

According to Worden et al. (33), the application of a geometric technique guided by anatomical landmarks can optimize inguinal lymph node identification, increasing surgical safety. Thus, the anatomical landmarks that can be considered in inguinal lymph node excision in dogs form the center of a triangle that can be previously delimited by connecting the papilla of the inguinal mammary gland, the base of the pectineus muscle, and the midline of the shaft of the penis in males, or the midline between the inguinal mammary glands (33).

For inguinal lymphadenectomy, the patient should be in dorsal recumbency and pelvic limbs must be abducted and extended caudally to expose the inguinal region. After visual and palpable delimitation of the three previously mentioned anatomical points, the inguinal lymph node is found in the most central region, surrounded by adipose tissue in the inguinal region and adjacent to the external pudendal artery and vein where it can be removed after ligation of its afferent and efferent vessels (33, 40).

## 3 Surgical approach: amputation

Amputation is a common procedure in canine and feline clinical practice, particularly indicated for appendicular bone neoplasms (41). It is often the only means of achieving wide tumor excision and remains the treatment of choice for local disease control and pain relief in dogs with OSA (5, 6, 42). A thorough preoperative assessment—including physical, neurological and orthopedic examination, a complete blood count, serum biochemical profile, urinalysis, and tumor staging—is essential when evaluating candidates for amputation. Although the procedure has relatively low morbidity and mortality, it is inherently invasive and traumatic, and could lead to significant blood loss (41).

Partial amputations are seldom recommended and should only be considered when prosthetic use is planned. Even in cases of distal neoplastic lesions, high-level amputation is preferred, as partial amputation without a prosthesis may result in excessive residual limb length, predisposing the patient to pressure ulcers and trauma (41). Intracapsular/intralesional amputation leaves residual tumor at the amputation site, while marginal amputation requires an excision the tumor near its pseudocapsule. A wide amputation removes normal bone tissue proximal to the tumor and adjacent soft tissues without an intervening joint between the amputation site and lesion. A radical amputation resects at least one joint proximal to the lesion, along with en bloc removal of involved soft tissues, ligaments, and tendons, establishing a natural anatomical barrier between the amputation site and tumor (41, 42).

OSA cells exhibit collagenolytic activity, degrading bone matrix through collagen breakdown. Within joints, this activity is partially inhibited by cartilage-derived collagenase inhibitors (43). Although joints are typically considered natural barriers to OSA invasion (44), tumor penetration remains possible, particularly in the coxofemoral joint. The ligament of the femoral head, which penetrates articular cartilage, may act as a conduit for neoplastic cells into the joint space. Additionally, soft tissue involvement (e.g., synovium, joint capsule) can facilitate invasion (41, 45).

Radical-margin amputation is the preferred surgical procedure for OSA due to its efficacy in local tumor control and analgesic benefits. However, even in patients without evidence of distant metastases, this intervention should be considered palliative rather than curative, given the high rate of distant metastases and the limited overall survival times reported in the literature, even with multimodal therapy. It may also serve as a palliative option for patients with macroscopic metastases or those with pathological fractures (5, 42, 65).

Thoracic limbs support 60% of body weight in dogs, while pelvic limbs bear 40% (46). Given the biomechanical implications of amputation, thorough patient evaluation and selection are critical (41, 47). A comprehensive preoperative assessment should include orthopedic and neurological examinations to assess limb function and identify any comorbidities; staging, which should involve orthogonal thoracic radiography or CT to detect pulmonary metastasis; and whole-body bone scintigraphy or positron emission tomography (PET) computed tomography (Pet-CT). Although not routinely used in clinical practice, bone scintigraphy-PET-CT offers high sensitivity for detecting skeletal metastases (42, 48). Special consideration is required for giant breeds, overweight dogs, and patients with orthopedic or neurological comorbidities, advanced degenerative joint disease, or prior limb surgeries. While these factors represent relative contraindications, such patients may still achieve favorable clinical outcomes with meticulous planning (42).

Several amputation techniques have been described for the surgical management of canine appendicular OSA in the thoracic limb. The most commonly performed procedures include shoulder disarticulation and forequarter amputation. Forequarter amputation, which involves removal of the entire limb including the scapula, is often preferred due to its technical efficiency, ability to achieve wide or radical margins, and shorter operative time (41). This technique has also demonstrated clinical success in managing extraskeletal OSA involving the brachial plexus (49). By contrast, shoulder disarticulation—a technique preserving the scapula—may lead to complications such as muscle atrophy and subsequent pressure injuries over bony prominences, particularly the acromion process and scapular spine (50). Distal amputations are less frequently employed but may be considered in limb-sparing contexts for distal lesions, where preserving proximal limb function remains feasible.

Similarly to thoracic limb amputation, various techniques have been described for pelvic limb amputation in dogs, with the most common being coxofemoral disarticulation, femoral diaphyseal osteotomy, and en bloc amputation with acetabulectomy or hemipelvectomy. Coxofemoral disarticulation is the most frequently performed technique, likely due to its technical simplicity. *En bloc* amputation with acetabulectomy or hemipelvectomy may be necessary to achieve wide surgical margins for proximal femoral tumors and naturally involvement of the pelvis (51). However, hemipelvectomy is the preferred approach for lesions involving the femoral head and neck, as it ensures adequate tumoral resection while addressing the anatomical complexity of the region (41, 47).

In a study involving 32 dogs undergoing thoracic limb amputation and 32 dogs undergoing pelvic limb amputation, 91% of owners reported no change in the attitude of the dogs postoperatively, and 88% noted complete or near-complete recovery of quality of life. Additionally, 73% of patients maintained their preoperative leisure activity levels and minor complications (e.g., surgical site infection and seroma) occurring in 8 patients (13%) (65).

Considering the amputation techniques described above, adhering to an exact sequence of dissection and resection is not the most critical factor for surgical success. The primary objective of amputation is limb removal with secure hemostasis (41), while minimizing additional harm to the patient. Achieving this requires thorough anatomical knowledge, appropriate analgesia, careful tissue handling, and meticulous wound closure. Arteries and veins should be individually identified, dissected, and ligated sutures (41). For larger-caliber vessels, double ligatures combined with transfixation sutures are advisable, particularly for arterial structures. Furthermore, literature recommends ligating efferent veins first during tumor resection to reduce the risk of intraoperative metastasis. By occluding venous outflow, tumor cells are less likely to enter systemic circulation, potentially mitigating the release of tumor emboli (52). However, it is important to note that this study was conducted in humans with non-small cell lung cancer, not in dogs with OSA.

### 3.1 Thoracic limb amputation

To perform the high thoracic limb amputation technique, the patient should be positioned in lateral recumbency, with the affected limb placed uppermost. The surgical site is clipped and aseptically prepared to establish a sterile field.

**Figure 1:**
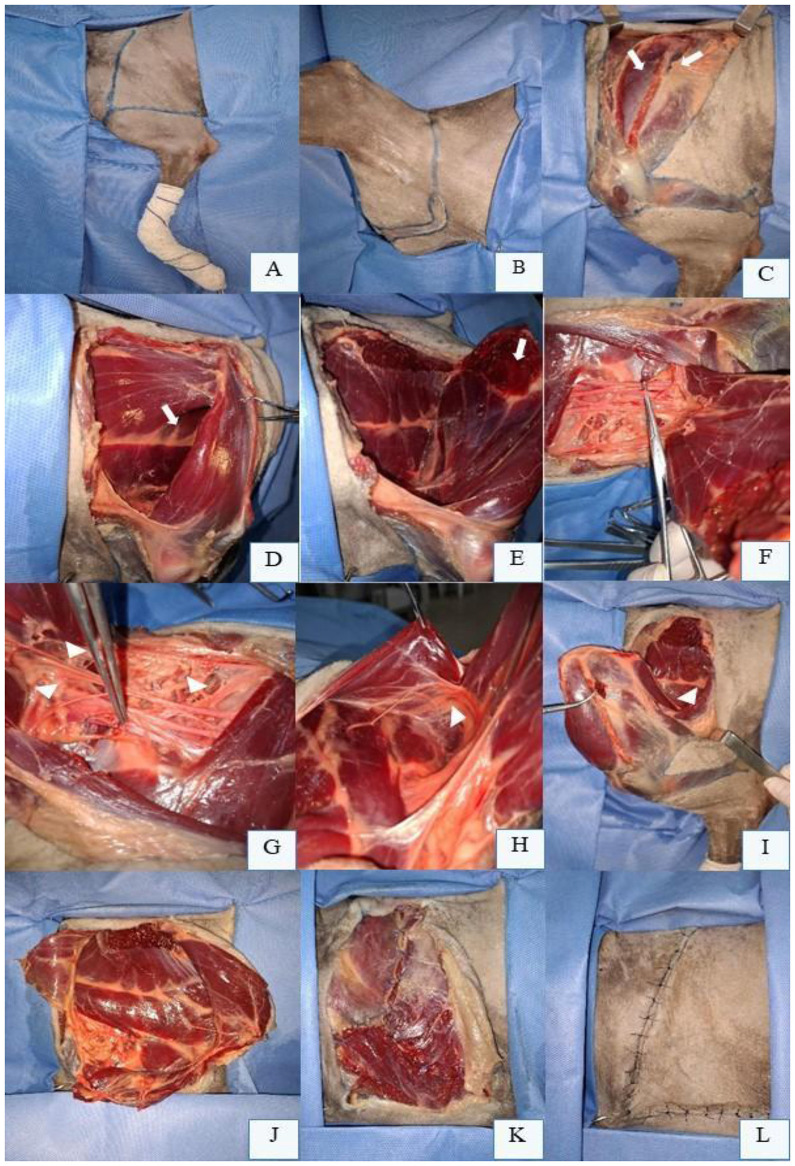
Left thoracic limb (LTL) amputation in a canine cadaver positioned in right lateral recumbency. **(A)** Preoperative skin incision marking from the scapular spine to the proximal third of the humerus, encircling the LTL. **(B)** Retracted skin edges in the axillary region, medial aspect of the LTL. **(C)** Transection of the trapezius muscle portions (white arrows) at their insertions on the scapular spine. **(D)** Scapular abduction using a Backhaus clamp, revealing the *serratus ventralis* muscle (white arrow) inserted medially on the scapula. **(E)** *Serratus ventralis* muscle separated from the costal surface of the scapula (white arrow). **(F)** Dissection and isolation of the axillary artery with a Mixter forceps on the ventromedial aspect of the scapula. **(G)** Axillary artery with double ligation and transfixation using 3-0 absorbable monofilament suture (Debakey forceps), alongside brachial plexus nerves (arrowheads). **(H)** Lateral thoracic vessels between the *latissimus dorsi* muscle (grasped by Debakey forceps) and deep pectoral muscle. **(I)** Cranial retraction of the scapula, transection of the *latissimus dorsi* muscle (arrowhead) near its aponeurosis, adjacent to the *triceps brachii* and teres major muscles. **(J)** Preserved extrinsic muscles after transection of the superficial and deep pectoral muscles ventrally for LTL removal. **(K)** Myorrhaphy of the pectoral muscles, *latissimus dorsi*, and *trapezius* muscles, protecting axillary vessel ligations. **(L)** Final L-shaped skin closure following LTL amputation.

The first technique to be described is high thoracic limb amputation (forequarter amputation), which involves removal of the scapula. The procedure begins with a skin incision proximal to the scapula, extending ventrally over the scapular spine to the level of the humeral greater tubercle (Figure 1A). The incision is then continued circumferentially around the limb at the axillary region (Figure 1B). To minimize the risk of cutaneous ischemic necrosis, incisions should be gently rounded rather than sharply angled, resulting in an inverted T- or L-shaped surgical wound upon closure. An alternative approach employs an elliptical incision extending from the proximal scapula to the axillary region (41, 53). Subsequent blunt dissection of the subcutaneous tissue allows elevation of the dorsal skin flap cranially above the scapula and caudally to the caudal border of the teres major muscle. Dissection of the fascia caudal to the teres major and the long head of the *triceps brachii* exposes the *latissimus dorsi* muscle (41, 47).

Following subcutaneous dissection, muscle resection begins. The cervical and thoracic portions of the *trapezius* muscle are transected at their insertions on the scapular spine (Figure 1C). The *omotransversarius* muscle is sectioned near the acromion, and the *cleidobrachialis* muscle is divided ventrally. The suprascapular artery (a branch of the superficial cervical artery) and cephalic vein, located cranially deep to the *cleidobrachialis*, are ligated with double sutures or transfixation ligatures before transection. The omobrachial vein, superficial to its homonymous muscle, is similarly ligated (41).

External rotation, lateral traction, or abduction of the scapula—facilitated by a Backhaus clamp on the proximal scapular spine—exposes the *rhomboideus* and *serratus ventralis* muscles (Figure 1D). These muscles are either elevated from their dorsal scapular insertions using a periosteal elevator or transected near the dorsal scapular border (Figure 1E). Subsequent scapular abduction reveals the brachial plexus and axillary vessels within the medial fascia (Figure 1F), which are individually ligated and resected (Figure 1G). The plexus may be locally blocked (e.g., 2 mg/kg bupivacaine) prior to distal transection.

Lateral thoracic vessels and nerves are identified between the *latissimus dorsi* and deep pectoral muscles (Figure 1H). Retracting the scapula cranially tenses the *latissimus dorsi*, which is then transected near its aponeurosis adjacent to the *triceps brachii* (Figure 1I). Associated vessels are ligated, and nerves may be blocked before resection. The external lateral thoracic artery, located cranially to the superficial pectoral muscle, is ligated and resected. Finally, the descending and transverse portions of the superficial pectoral muscle and deep pectoral muscle are transected ventrally (Figure 1J). Closure proceeds with layered suturing to ensure tension-free apposition (41, 47).

Thorough hemostasis must be ensured prior to closure. The surgical site is lavaged with warm sterile saline to remove debris and reduce bacterial load. Muscle layers are apposed by suturing the fascia of transected muscles (Figure 1K). The cervical portion of the *trapezius* muscle is sutured to its thoracic component dorsally, while the *omotransversarius, brachiocephalicus*, and ventral border of the cervical *trapezius* are sutured to the *latissimus dorsi*. The pectoral muscles are anchored dorsally to the ventral edge of the *latissimus dorsi* and *brachiocephalicus* to shield the axillary vessel ligatures. In the subcutaneous layer, dead space should be meticulously minimized to prevent seroma formation; a closed suction drain may be placed if necessary. Skin closure is achieved via intradermal or cutaneous sutures, typically in an inverted T- or L-shaped configuration (Figure 1L) (41, 53).

The shoulder disarticulation technique for thoracic limb amputation is similar to forequarter amputation but preserves the scapula by isolating the glenohumeral joint. A circumferential skin incision is made around the proximal third of the humerus to access the joint. Unlike forequarter amputation, the axillary vessels are not ligated; however, the brachial artery and vein require double ligation, and nerves are transected at this level following limb abduction prior to joint disarticulation (53, 54). This technique is less commonly performed than forequarter amputation, which removes the entire scapula and limb segments. Preservation of the scapula often leads to long-term muscular atrophy around the retained bone (53).

### 3.2 Pelvic limb amputation

**Figure 2:**
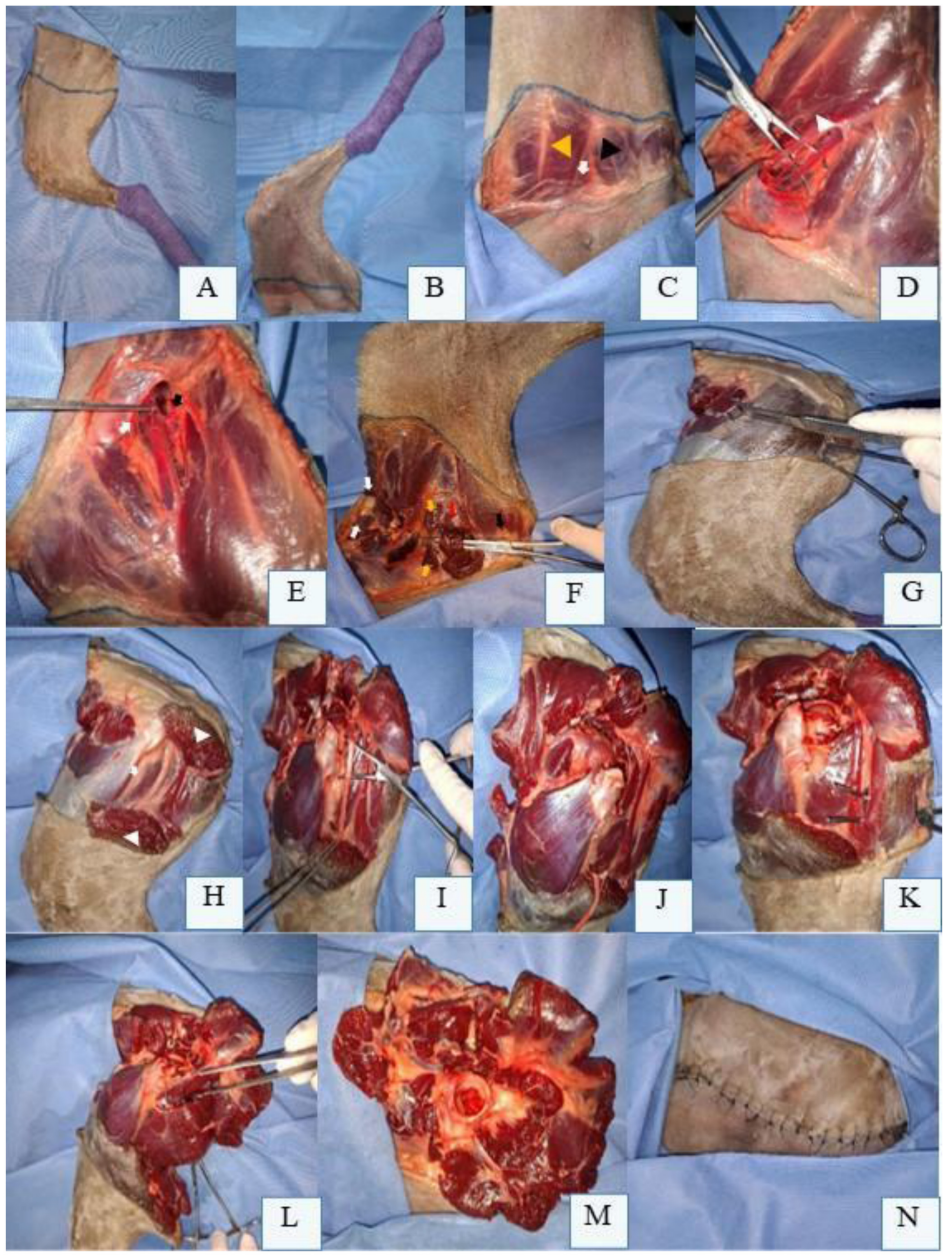
Preoperative skin incision marking for left pelvic limb (LPL) amputation in a canine cadaver positioned in right lateral recumbency. **(A)** Lateral aspect of the LPL with skin marking along the proximal third of the femur, extending from the inguinal fold to the ischial tuberosity. **(B)** Medial aspect of the LPL with skin marking along the proximal third of the femur. **(C)** Abducted LPL revealing the femoral triangle (white arrow) beneath the deep femoral fascia, bounded by the *pectineus* muscle (black arrowhead) and *sartorius* muscle (yellow arrowhead). **(D)** Dissection and isolation of the femoral artery, femoral vein, and saphenous nerve (over Mixter forceps) within the femoral triangle (arrowhead) after opening the deep femoral fascia. **(E)** Femoral artery and vein ligated proximal to the origin of the superficial circumflex iliac (white arrow) and lateral circumflex femoral branches (black arrow). **(F)** Transection of the cranial and caudal portions of the *sartorius* muscle (white arrows), *pectineus* muscle (yellow arrows), and *gracilis* muscle (black arrow) ventrally. The *adductor magnus* muscle (under the *semimembranosus* muscle, red arrow) is isolated and transected. **(G)** *Biceps femoris* muscle isolated and incised ventrally. **(H)** *Biceps femoris* muscle reflected to expose the femoral greater trochanter (white arrow) and sciatic nerve (red arrow). **(I)** Sciatic nerve dissected and isolated with Mixter forceps prior to transection. **(J)** *Articularis coxae* muscle cranial to the coxofemoral joint (black arrow). **(K)** *Semitendinosus* muscle isolated for transection ventrally. **(L)** *Adductor longus* muscle isolated for transection. **(M)** Remaining cranial muscles sutured to caudal muscles to protect femoral vessel ligations and create a muscular cushion over the acetabulum. **(N)** Final skin closure after myorrhaphy and dead space obliteration.

The coxofemoral disarticulation technique for pelvic limb amputation in dogs begins with the patient positioned in lateral recumbency, affected limb uppermost, clipped and aseptically prepared. A semicircular skin incision is initiated cranially at the inguinal fold, extending along the proximal third of the femur to the ischial tuberosity (Figure 2A). The lateral aspect of the incision is made slightly more distal than the medial aspect (Figure 2B) to optimize tension-free closure.

The limb is abducted to expose the deep medial femoral fascia (Figure 2C), which is dissected to identify the femoral triangle bounded by the *sartorius* and *pectineus* muscles. Within this triangle, the femoral artery, femoral vein, and saphenous nerve are isolated (Figure 2D). When feasible, these vessels are individually ligated with double sutures and transfixation ligatures proximal to the origin of the superficial circumflex iliac and lateral circumflex femoral branches (Figure 2E). Alternatively, each branch is ligated separately. The medial circumflex femoral artery and vein, located caudally within the femoral triangle, are similarly ligated and transected. The saphenous nerve should be blocked with local anesthetic (e.g., 2 mg/kg bupivacaine) before transection (41, 47). With the limb abducted, the cranial and caudal portions of the *sartorius, rectus femoris, pectineus*, and *gracilis* muscles are transected ventrally. Deep to the *gracilis*, the *adductor magnus* muscle is divided. Following limb adduction, dissection proceeds to the lateral aspect (Figure 2F), ensuring complete removal of the limb while preserving neurovascular structures and minimizing soft tissue trauma.

On the lateral aspect of the limb, the skin is reflected dorsally to expose the *fascia lata* and superficial periarticular hip muscles, including the *tensor fasciae latae* cranially and the *biceps femoris* caudally (41, 53, 55). The *tensor fasciae latae* is sectioned at its fascial portion, while the *biceps femoris* is incised proximally along ventral portion (Figure 2G) and reflected dorsally to expose the femoral greater trochanter. At this stage, the superficial gluteal, middle gluteal, deep gluteal, and *piriformis* muscles are identified. Among these, only the superficial gluteal muscle inserts on the third trochanter of the femur; the others attach to the femoral greater trochanter and are transected near their insertions. Reflecting the superficial gluteal and *piriformis* muscles exposes the caudal gluteal artery and vein and the sciatic nerve (Figure 2H), which course parallel to one another. The vessels are individually ligated, and the nerve is dissected free (Figure 2I) before being blocked with local anesthetic and transected (41, 47).

The *gemelli* muscles, located medially to the femoral greater trochanter, are transected ventrally. Deep to these, the external obturator muscle is sectioned, followed by the internal obturator muscle at its tendons of origin. The *articularis coxae* muscle, closely associated with the cranial aspect of the coxofemoral joint capsule (Figure 2J), is incised cranially. A branch of the lateral circumflex femoral artery is often observed in this region. On the lateral thigh, caudal to the pelvic limb, the *semitendinosus* and *semimembranosus* muscles are incised ventrally (Figure 2K). Subsequently, the *adductor longus* muscle is similarly transected (Figure 2L) (41, 47, 53).

The limb is abducted again to identify remaining muscles on the medial aspect near the hip joint. The *iliopsoas* muscle, associated with the femoral nerve, is transected ventrally or tendon of origin on the femoral lesser trochanter, and the nerve is blocked (e.g., 2 mg/kg bupivacaine) before transection. Caudally, the *adductor longus* and *quadratus femoris* muscles are identified and incised ventrally. The joint capsule incision is extended medially and ventrally. If intact, the ligament of the femoral head is transected using a No. 11 scalpel blade or Metzenbaum scissors, fully freeing the pelvic limb (41, 47, 53).

After confirming adequate hemostasis, the surgical site is lavaged with warm sterile saline. Remaining cranial muscles are sutured to caudal muscles to protect femoral vessel ligations (Figure 2M). The *biceps femoris* fascia is opposed to the *gracili*s and *semitendinosus* muscles, while the tensor fasciae latae is sutured to the *sartorius* and *iliopsoas* muscles using a continuous or interrupted suture pattern. Dead space should be meticulously closed, and a surgical drain may be placed if necessary. Routine intradermal or cutaneous sutures are then performed to complete the closure (Figure 2N) (41, 47, 53).

Complications associated with amputations are uncommon, with overall rates of 13% (65) including surgical site infections and seroma. An association between surgical site infections and increased survival time was found in patients undergoing limb-sparing procedures (42, 56), though this association was not observed in amputated patients (57). Other potential complications include intraoperative hemorrhage, air emboli, accidental access into the thoracic cavity during high thoracic limb amputation, and phantom limb syndrome (47). Neuroma may present as focal pain at the surgical site. A rarely reported complication is intervertebral disc herniation; Séguin et al. (41) hypothesize that increased cervical range of motion in dogs—particularly after thoracic limb amputation—places additional stress on the cervical spine, elevating the risk of disc herniation (65).

Postoperative care following amputation must prioritize analgesia and include cold compress application to the surgical site (10–15 min every 8 h for 3–4 days). Patients should be housed on non-slip flooring during the initial recovery phase to aid adaptation, with short leash walks recommended for approximately 15 days. Most dogs adapt within a mean period of 4 weeks, and the majority ambulate effectively by the day following surgery, including large breeds (41, 42, 47).

### 3.3 Hemipelvectomy

Hemipelvectomy is a surgical technique involving the resection of a pelvic segment, which may include an entire hemipelvis (59). It is primarily indicated for the removal of malignant neoplasms affecting the pelvic region or adjacent soft tissues (58). This procedure demands rigorous patient selection, as orthopedic or neurological comorbidities may compromise postoperative ambulation (51). Successful outcomes rely on thorough anatomical knowledge of the pelvis, associated musculature, and neurovascular structures; adherence to surgical oncology principles; appropriate closure techniques; and robust perioperative analgesia and postoperative care. Due to the technique's complexity and invasiveness, meticulous planning and execution are critical to minimizing complications and optimizing patient recovery (25).

Comprehensive tumor staging is essential for hemipelvectomy to determine the presence or absence of distant metastasis. While radiography aids in the initial diagnosis of pelvic neoplasia, it does not provide sufficient detail to assess local tumor extent. Pelvic CT is imperative for precisely delineating tumor location and invasion, facilitating optimal surgical planning for OSA in this anatomical site (25, 51).

Critical challenges in performing hemipelvectomy include the anatomical complexity of the region, which involves associated muscle groups, neurovascular bundles, and abdominal/pelvic organs—notably the urethra. Adequate closure of the surgical defect is paramount and may utilize preserved residual musculature or, if extensive resection is required, polypropylene mesh reconstruction to ensure complete tumor removal (25).

**Figure 3:**
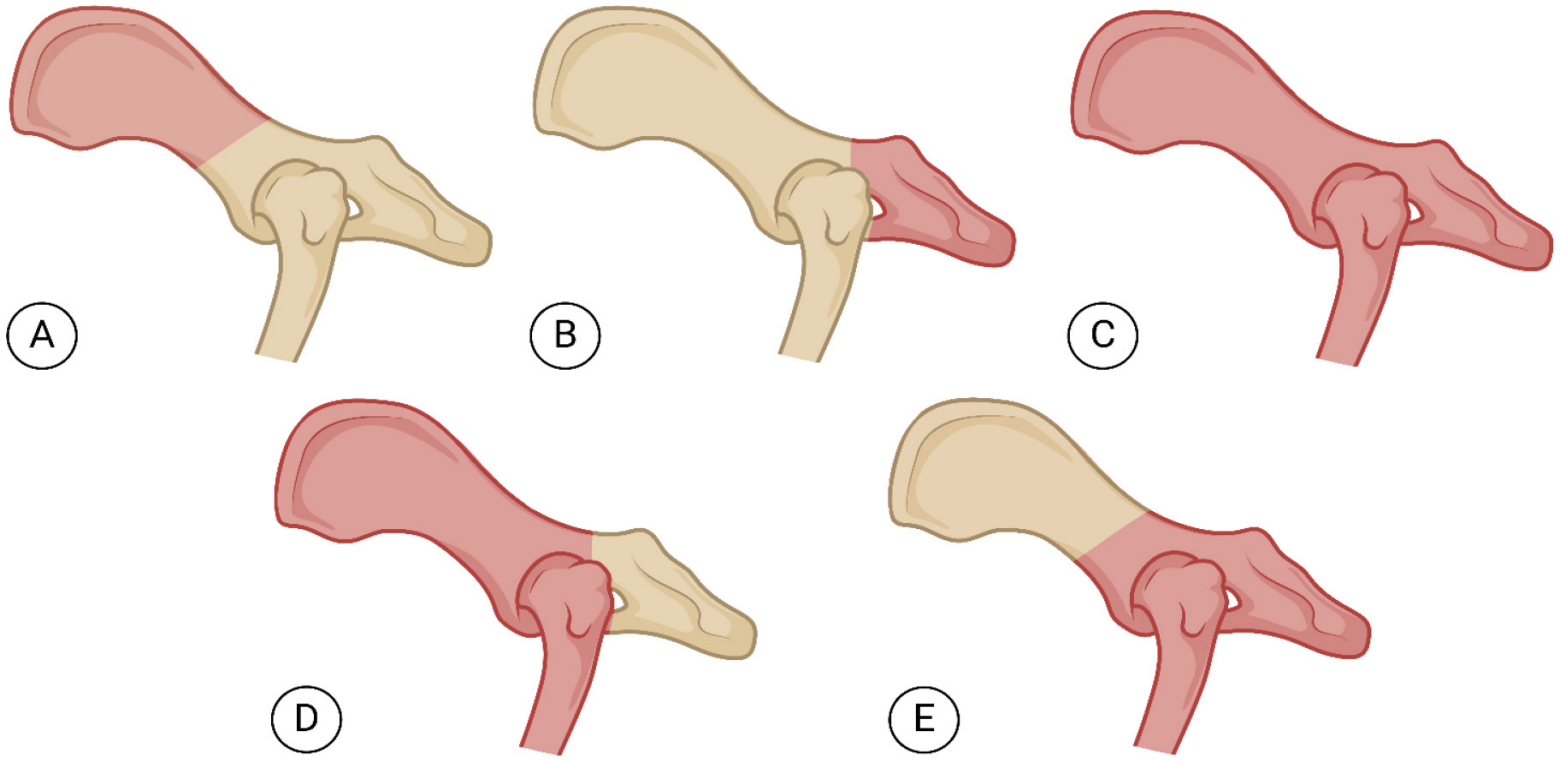
Lateral view of the pelvis illustrating (in pink) diferent modalities of canine hemipelvectomy. **(A)** Cranial internal hemipelvectomy (ileectomy): Resection of the ilium while preserving the sacrum and acetabulum. **(B)** Caudal internal hemipelvectomy (ischiectomy): Removal of the ischium of a hemipelvis. **(C)** Total hemipelvectomy: Total removal of one hemipelvis and the remainder of the ipsilateral pelvic limb. **(D)** Cranial external hemipelvectomy: Romoval of the ilium, acetabulum, and pubis to the pelvic symphysis with limb amputation. **(E)** Caudal external hemipelvectomy: Removal of the acetabulum, body of the ilium from the sacroiliac junction, ipsilateral pubis and ischium and medially to the level of the pelvic symphysis, plus the remainder of the ipsilateral pelvic limb. Figure created using BioRender^®^.

**Figure 4:**
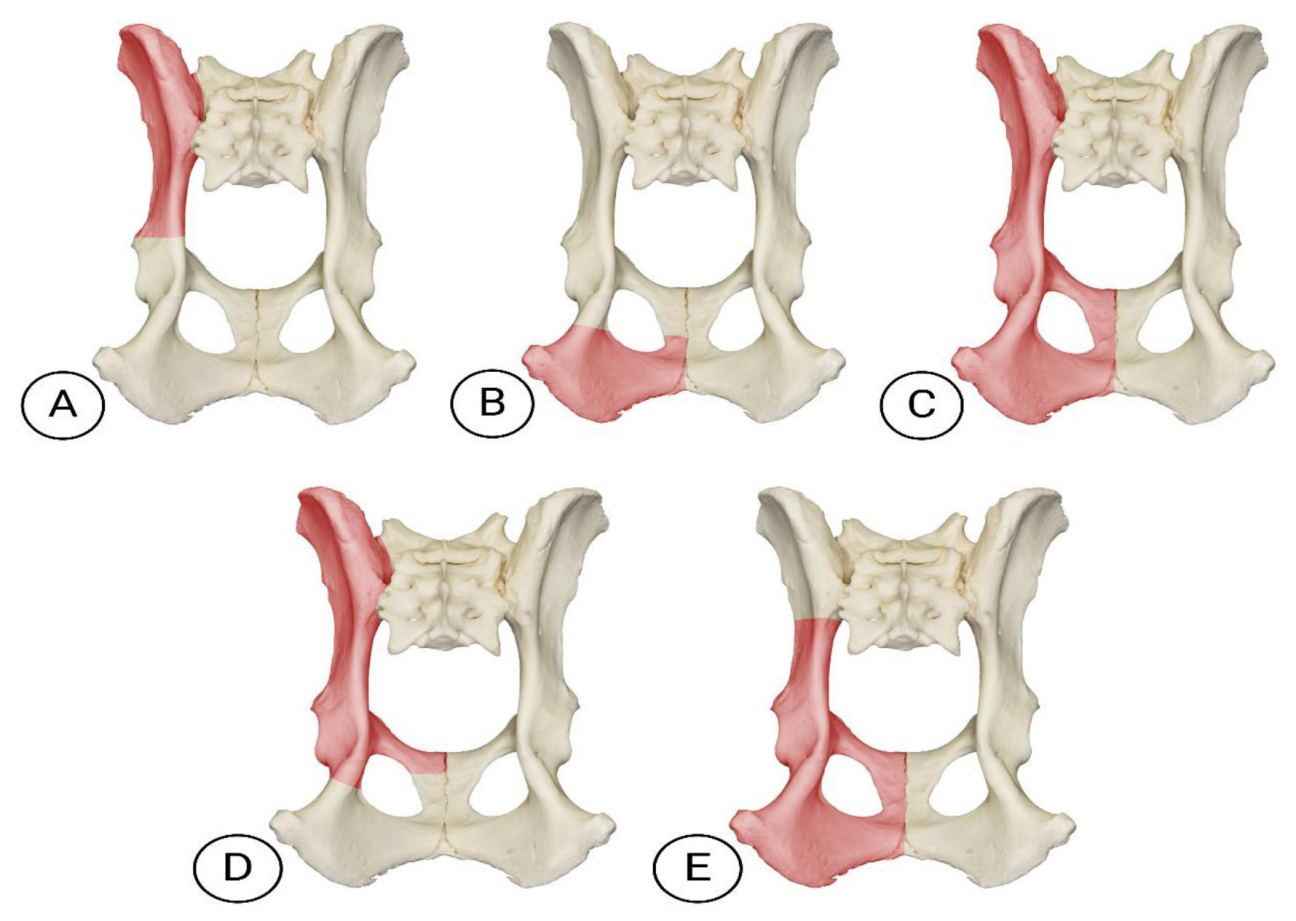
Ventral view of the pelvis illustrating (in pink) different modalities of canine hemipelvectomy. **(A)** Cranial internal hemipelvectomy (ileectomy): Resection of the ilium while preserving the sacrum and acetabulum. **(B)** Caudal internal hemipelvectomy (ischiectomy): Removal of the ischium of a hemipelvis. **(C)** Total hemipelvectomy: Total removal of one hemipelvis and the remainder of the ipsilateral pelvic limb. **(D)** Cranial external hemipelvectomy: Romoval of the ilium, acetabulum, and pubis to the pelvic symphysis with limb amputation. **(E)** Caudal external hemipelvectomy: Removal of the acetabulum, body of the ilium from the sacroiliac junction, ipsilateral pubis and ischium and medially to the level of the pelvic symphysis, plus the remainder of the ipsilateral pelvic limb. Photo adapted from courtesy of www.skullsunlimited.com.

The pelvic limb can be preserved in select hemipelvectomy techniques, provided the weight-bearing axis remains unaffected by surgical resection. Variations in technique (Figures 3, 4) depend on tumor type and anatomical location (58). Hemipelvectomy is broadly classified as internal or external, following terminology from human medicine. Internal hemipelvectomy preserves the limb distal to the femur while resecting a pelvic segment, whereas external hemipelvectomy involves amputation of the ipsilateral limb alongside the hemipelvis (58).

Internal hemipelvectomy is subdivided into cranial internal (resection of the ilium, including its wing and body, termed unilateral iliectomy) and caudal internal (resection of the ischium, termed unilateral ischiectomy). External hemipelvectomy includes total external (removal of the entire hemipelvis and limb), cranial external (resection of the ilium, acetabulum, and pubis to the pelvic symphysis with limb amputation), and caudal external (resection of the acetabulum, body of the ilium from the sacroiliac junction, ipsilateral pubis and ischium and medially to the level of the pelvic symphysis, plus the remainder of the ipsilateral pelvic limb acetabulum, ilium, pubis, and ischium with limb removal). A midline hemipelvectomy (acetabulectomy with limb amputation) is also described but less commonly utilized (25, 58). In veterinary medicine, classifications are often simplified into total hemipelvectomy, considering entire hemipelvis and limb removal via sacroiliac disarticulation and pelvic symphysis osteotomy, and subtotal hemipelvectomy (25, 54, 58). Subtotal procedures are further categorized as mid-cranial (ilium and acetabulum resection), mid-caudal (ischium and acetabulum resection), or caudal (ischium-only resection). Limb preservation is feasible only in subtotal caudal hemipelvectomy, as the acetabulum remains intact (25, 54).

Technique selection requires meticulous preoperative planning to prioritize clean surgical margins and preserve neurovascular structures critical to limb function when applicable. This balance ensures oncological efficacy while optimizing postoperative mobility and quality of life (25).

**Figure 5:**
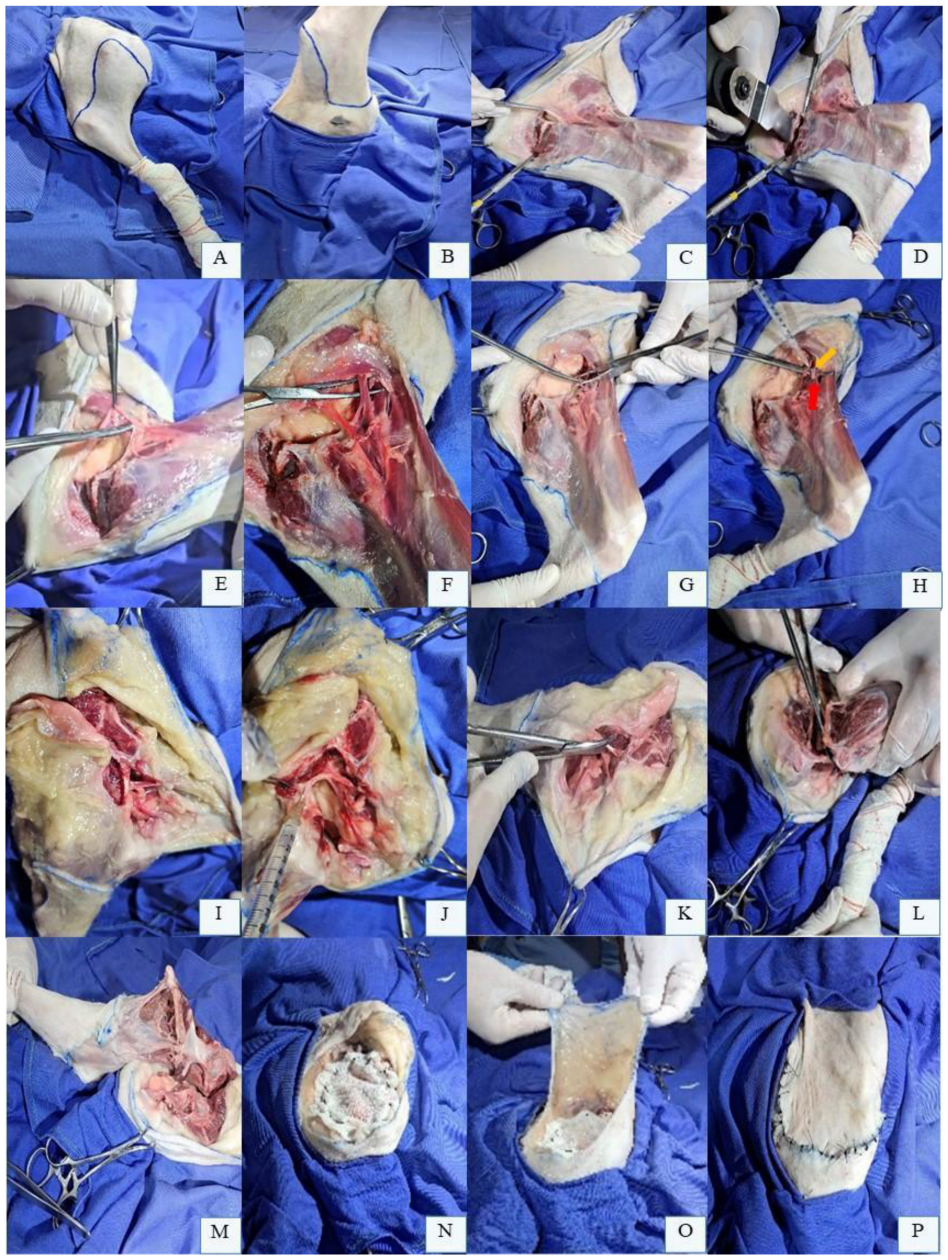
Total hemipelvectomy in a canine cadaver positioned in right lateral recumbency. **(A)** Preoperative skin incision planning for creation of an inguinal fold skin flap, marked with a surgical skin marker on the lateral aspect of the left pelvic limb (LPL). **(B)** Skin incision marked on the medial aspect of the LPL. **(C)** Exposure of the pelvic symphysis for detachment of prepubic tendons and muscles originating from the pelvic symphysis. **(D)** Use of a malleable retractor to protect pelvic cavity organs during pelvic symphysis osteotomy with an oscillating saw. **(E)** Soft tissue along the pelvic border is released via sharp dissection and blunt separation. **(F)** Dissection and isolation of the external iliac artery and vein. **(G)** Double ligation of the external iliac artery and vein prior to their bifurcation. **(H)** Local anesthetic infiltration of the femoral nerve (yellow arrow), located deep to the transected iliopsoas muscle (red arrow). **(I)** On the lateral thigh, the thoracolumbar fascia and cranial epaxial muscles medial to the iliac wing are separated. **(J)** Sciatic nerve is infiltrated with local anesthetic prior to transection. **(K)** Dorsocaudal muscles and sacrotuberous ligament are transected near the sacrum. **(L)** Caudally, pelvic diaphragm muscles are transected close to the ischium to preserve pudendal vessels and their branches. **(M)** LPL freed following sacroiliac joint disarticulation. **(N)** Abdominal wall reconstruction using the greater omentum and polypropylene mesh. **(O)** Transposition of the inguinal fold skin flap over the surgical defect. **(P)** Final appearance of skin closure after total hemipelvectomy.

For total hemipelvectomy, the entire pelvic limb, dorsal midline, and ventral regions of the pelvis and caudal abdomen are clipped and aseptically prepared. The patient is positioned in lateral recumbency with the affected limb uppermost. A surgical marker outlines the incision on the lateral and medial thigh, converging to ensure tension-free closure. To create a skin flap from the inguinal fold, the incision is marked from the ischial tuberosity, extending laterally over the greater trochanter and descending to the patellar tendon (Figure 5A). Medially, the incision ascends to the *pectineus* muscle and meets the lateral mark at the ischial tuberosity (Figure 5B).

The procedure begins with a medial approach: the limb is abducted, and the marked skin and subcutaneous tissue are dissected and reflected to expose the pelvic symphysis. A periosteal elevator detaches the prepubic tendons and muscles originating from the pelvic symphysis (Figure 5C). Under constant saline irrigation, an oscillating saw performs an osteotomy of the pelvic symphysis, while a malleable retractor protects the urethra and rectum (Figure 5D). Subsequent dissection along the pelvic border releases all soft tissues (Figure 5E), exposing the external iliac artery and vein. These vessels are isolated (Figure 5F), double-ligated individually with 3-0 absorbable monofilament suture (Figure 5G), and divided proximal to their femoral bifurcation. With the limb abducted, the *iliopsoas* muscle is infiltrated with local anesthetic, transected incrementally, and the underlying femoral nerve is blocked before transection (Figure 5H). Cranially, dissection proceeds along the iliac wing, thoracolumbar fascia, and epaxial muscles medial to the ilium (Figure 5I), reaching the sacroiliac joint. This joint is exposed by transecting the dorsolateral sacrocaudal and *longissimus lumborum* muscles. Dorsomedial dissection along the iliac body identifies the sciatic nerve, which is blocked and transected (Figure 5J). The dorsocaudal muscles and sacrotuberous ligament are sectioned near the sacrum (Figure 5K), while the pelvic diaphragm muscles are divided close to the ischium to preserve pudendal vessels (Figure 5L).

After exposing the sacroiliac joint (Figure 5M), disarticulation is performed with an oscillating saw or osteotome. Prior to closure, hemostasis is meticulously confirmed. Abdominal wall reconstruction using greater omentum and polypropylene mesh (Figure 5N) is indicated if the sartorius muscle cannot be spared. The inguinal skin flap is transposed to the defect (Figure 5O), sutured to the recipient bed (Figure 5P), and a closed suction drain is placed to minimize seroma formation.

Reported complications in dogs and cats undergoing hemipelvectomy include intraoperative hemorrhage, iatrogenic urethral injury, abdominal incisional hernia, surgical site discharge, and wound dehiscence (25, 51). Preventive measures such as intraoperative blood bank availability, urethral or rectal catheterization, and temporary purse-string suture of the anus are recommended to mitigate risks (25).

## 4 Conclusion

Amputation procedures are recommended for local control, which is the main objective in the therapeutic management of osteosarcoma. It involves the surgical excision of an appendicular segment, with consequent redistribution of loads and overloading of the remaining limbs. Therefore, individual and careful selection of the patient based on the general assessment and the muscular, skeletal and nervous systems, associated with anatomical knowledge, are essential for a favorable outcome.
